# Host KIR/HLA-C Genotypes Determine HIV-Mediated Changes of the NK Cell Repertoire and Are Associated With Vpu Sequence Variations Impacting Downmodulation of HLA-C

**DOI:** 10.3389/fimmu.2022.922252

**Published:** 2022-07-15

**Authors:** Sarah Vollmers, Annabelle Lobermeyer, Annika Niehrs, Pia Fittje, Daniela Indenbirken, Jacqueline Nakel, Sanamjeet Virdi, Sebastien Brias, Timo Trenkner, Gabriel Sauer, Sven Peine, Georg M.N. Behrens, Clara Lehmann, Anja Meurer, Ramona Pauli, Nils Postel, Julia Roider, Stefan Scholten, Christoph D. Spinner, Christoph Stephan, Eva Wolf, Christoph Wyen, Laura Richert, Paul J. Norman, Jürgen Sauter, Alexander H. Schmidt, Angelique Hoelzemer, Marcus Altfeld, Christian Körner

**Affiliations:** ^1^ Leibniz Institute of Virology, Hamburg, Germany; ^2^ First Department of Medicine, Division of Infectious Diseases, University Medical Center Hamburg-Eppendorf, Hamburg, Germany; ^3^ Department I for Internal Medicine, University Hospital of Cologne, Cologne, Germany; ^4^ Institute for Transfusion Medicine, University Medical Center Hamburg-Eppendorf, Hamburg, Germany; ^5^ Department for Rheumatology and Clinical Immunology, Hannover Medical School, Hannover, Germany; ^6^ Department I for Internal Medicine, Division of Infectious Diseases, University Hospital Cologne, Cologne, Germany; ^7^ German Center for Infection Research (DZIF), Partner Site Bonn-Cologne, Cologne, Germany; ^8^ Center for Molecular Medicine Cologne, University of Cologne, Cologne, Germany; ^9^ Center for Internal Medicine and Infectiology, Munich, Germany; ^10^ Medizinisches Versorgungszentrum (MVZ) am Isartor, Munich, Germany; ^11^ Prinzmed, Practice for Infectious Diseases, Munich, Germany; ^12^ Department of Internal Medicine IV, Department of Infectious Diseases, Ludwig-Maximilians University Munich, Munich, Germany; ^13^ German Center for Infection Research (DZIF), Partner Site Munich, Munich, Germany; ^14^ Praxis Hohenstaufenring, Cologne, Germany; ^15^ Technical University of Munich, School of Medicine, University Hospital rechts der Isar, Department of Internal Medicine II, Munich, Germany; ^16^ Infectious Diseases Unit, Goethe-University Hospital Frankfurt, Frankfurt, Germany; ^17^ MUC Research, Munich, Germany; ^18^ Praxis am Ebertplatz, Cologne, Germany; ^19^ University of Bordeaux, Inserm U1219 Bordeaux Population Health, Inria Sistm, Bordeaux, France; ^20^ Division of Biomedical Informatics and Personalized Medicine, University of Colorado, Aurora, CO, United States; ^21^ Department of Immunology and Microbiology, University of Colorado, Aurora, CO, United States; ^22^ DKMS, Tübingen, Germany; ^23^ DKMS Life Science Lab, Dresden, Germany; ^24^ German Center for Infection Research (DZIF), Partner Site Hamburg-Lübeck-Borstel-Riems, Hamburg, Germany

**Keywords:** NK cell, KIR, HLA-C, HIV-1, Vpu

## Abstract

NK cells play a pivotal role in viral immunity, utilizing a large array of activating and inhibitory receptors to identify and eliminate virus-infected cells. Killer-cell immunoglobulin-like receptors (KIRs) represent a highly polymorphic receptor family, regulating NK cell activity and determining the ability to recognize target cells. Human leukocyte antigen (HLA) class I molecules serve as the primary ligand for KIRs. Herein, HLA-C stands out as being the dominant ligand for the majority of KIRs. Accumulating evidence indicated that interactions between HLA-C and its inhibitory KIR2DL receptors (KIR2DL1/L2/L3) can drive HIV-1-mediated immune evasion and thus may contribute to the intrinsic control of HIV-1 infection. Of particular interest in this context is the recent observation that HIV-1 is able to adapt to host *HLA-C* genotypes through Vpu-mediated downmodulation of HLA-C. However, our understanding of the complex interplay between *KIR/HLA* immunogenetics, NK cell-mediated immune pressure and HIV-1 immune escape is still limited. Therefore, we investigated the impact of specific *KIR/HLA-C* combinations on the NK cell receptor repertoire and HIV-1 Vpu protein sequence variations of 122 viremic, untreated HIV-1^+^ individuals. Compared to 60 HIV-1^-^ controls, HIV-1 infection was associated with significant changes within the NK cell receptor repertoire, including reduced percentages of NK cells expressing NKG2A, CD8, and KIR2DS4. In contrast, the NKG2C^+^ and KIR3DL2^+^ NK cell sub-populations from HIV-1^+^ individuals was enlarged compared to HIV-1^-^ controls. Stratification along *KIR/HLA-C* genotypes revealed a genotype-dependent expansion of KIR2DL1^+^ NK cells that was ultimately associated with increased binding affinities between KIR2DL1 and HLA-C allotypes. Lastly, our data hinted to a preferential selection of Vpu sequence variants that were associated with HLA-C downmodulation in individuals with high KIR2DL/HLA-C binding affinities. Altogether, our study provides evidence that HIV-1-associated changes in the KIR repertoire of NK cells are to some extent predetermined by host *KIR2DL/HLA-C* genotypes. Furthermore, analysis of Vpu sequence polymorphisms indicates that differential KIR2DL/HLA-C binding affinities may serve as an additional mechanism how host genetics impact immune evasion by HIV-1.

## Introduction

Natural killer (NK) cells are innate lymphocytes crucially involved in antiviral immunity and tumor surveillance (1, 2). Their main effector functions comprise production of pro-inflammatory cytokines and elimination of virus-infected and transformed cells through direct cytotoxicity (3, 4). NK cells utilize a large number of activating and inhibitory receptors to distinguish between healthy (“self”) and aberrant cells (“non-self”) (5). Inhibitory receptors mainly interact with various human leukocyte antigen (HLA) class I molecules, maintaining self-tolerance, whereas receptors with activating properties are able to recognize stress ligands on potential target cells. An important group of NK cell receptors are killer-cell immunoglobulin-like receptors (KIRs) that predominantly recognize HLA class I molecules. The most recently evolved HLA class I molecule is HLA-C, which is only present in humans and great apes (6). Contrary to HLA-A and HLA-B, virtually all HLA-C allotypes are recognized by KIRs, making HLA-C a dominant ligand for the regulation of NK cell activity (7, 8). Co-evolution of HLA class I and KIRs resulted in a remarkable diversity with distinct binding specificities between their members (8, 9). The inhibitory receptor KIR3DL2 binds only to HLA-A3/11 (10, 11), while KIR3DL1 recognition is limited to a subset of HLA-A and -B molecules that contain the serological motif Bw4 (12). In contrast, HLA-C is recognized by a number of inhibitory and activating KIRs. HLA-C molecules can be distinguished into two groups, defined by a dimorphism at position 80 in the α1 domain (7). HLA-C group 1 (HLA-C1) allotypes are characterized by an asparagine at position 80 and are predominantly recognized by the inhibitory receptors KIR2DL2 and KIR2DL3 as well as some allotypes of the activating receptor KIR2DS2 (13). *HLA-C* alleles encoding for group 2 (HLA-C2) molecules contain a lysine at position 80 and interact with the inhibitory KIR2DL1, the activating KIR2DS1 and a few KIR2DS5 allotypes (14–16). Additionally, certain KIR2DL2 and KIR2DL3 allotypes are cross-reactive with HLA-C2 molecules. Finally, KIR2DS4 is able to bind HLA-C1 and -C2 molecules with variegated affinities (17, 18).

Both, HLA-C and KIRs, are highly polymorphic, resulting in a large number of allotype combinations within a population that are further characterized by variegating binding affinities (8, 19). Accumulating evidence strongly indicates that interactions between KIRs and HLA-C impact the course of pathologic conditions and infectious diseases, such as preeclampsia (20), arthritis (21) and HCV infection (22). Most prominently, several studies have demonstrated a major impact of *HLA class I* genes in the outcome of HIV-1 infection, alone or in combination with certain *KIR*s (23–27). In recent years accumulating evidence suggests a role for HLA-C in immune control as well as HIV-1-associated immune escape. For one, increased HLA-C expression levels were associated with protection against HIV-1 progression (28). The ability of the accessory HIV-1 protein Vpu to downregulate HLA-C expression level on infected cells (29) and its adaption to *HLA-C* genotypes through sequence variations in Vpu was indicative of a novel HLA-C-associated immune evasion strategy (30). Further epidemiological and experimental evidence demonstrated that KIR^+^ NK cells can recognize HIV-1-mediated alterations of HLA-C expression (31) or changes in the HLA-C/peptide complex (32–37). However, the complex interplay between host genetics, NK cell-mediated immune pressure and HIV-1 immune escape is still only partially understood. In particular, the high allelic diversity of HLA-C and KIR2DL molecules and previous limitations in the resolution of *KIR* alleles have complicated the generation of models that would allow a clearer view of the mechanisms underlying the intrinsic control of HIV-1 and the contribution of KIR2DL and their HLA-C ligands.

In this study, we investigated the impact of KIR/HLA-C interactions on the NK cell repertoire and HIV-1 sequence polymorphisms in the context of HIV-1 infection. For this, we determined the binding affinities of various KIR2DL/HLA-C allotype combinations and conducted a comprehensive phenotypical characterization of NK cells from a cohort of viremic, untreated HIV-1^+^ individuals compared to HIV-1^-^ controls. Finally, we performed next-generation sequencing (NGS) of *Vpu* from matched plasma and PBMC samples from HIV-1^+^ individuals. Herein, our results indicated that the underlying host genetics influences the NK cell receptor repertoire in HIV-1^+^ individuals as well as the selection of Vpu sequence variants.

## Materials and Methods

### Human Subjects

Peripheral blood samples were obtained from healthy blood donors (n = 45) recruited at the University Medical Center Hamburg-Eppendorf, Hamburg, Germany. In addition, peripheral blood was obtained from anonymized healthy human donors (n = 15) at the Institute for Transfusion Medicine, University Medical Center Hamburg-Eppendorf, Hamburg, Germany. Information on age and sex was not available for all subjects, however was not relevant for the purpose of the study. Cryopreserved peripheral blood mononuclear cells (PBMC) and plasma samples from untreated HIV-1-infected individuals (n = 122) were obtained from the Translational Platform HIV (TP-HIV) Cohort by the German Center for Infection Research (DZIF).

### Sample Processing

Peripheral blood mononuclear cells (PBMCs) were isolated from peripheral blood from healthy human donors by density gradient centrifugation. Isolated PBMCs were cryopreserved for upcoming experiments in liquid nitrogen tanks in heat-inactivated fetal bovine serum (Sigma-Aldrich) supplemented with 10% (v/v) DMSO (Sigma-Aldrich). Cryopreserved PBMCs were thawed by adding PBMCs dropwise to complete RPMI-1640 medium (Life Technologies) supplemented with 10% (v/v) heat-inactivated FBS (Sigma-Aldrich), 100 U/ml penicillin, 100 μg/ml streptomycin (Sigma-Aldrich) and 25 U/ml Benzonase Nuclease (Merck Milipore Novagen). Thawed PBMCs were incubated for 30 min at 37°C and washed with PBS (Sigma-Aldrich) and then used for antibody staining and genomic DNA isolation.

### Cell Lines

HEK293T/17 cell line (ATCC, Cat#CRL-11268) was cultured in Dulbecco’s Modified Eagle Medium (DMEM, Life Technologies) supplemented with 10% (v/v) heat-inactivated FBS (Sigma-Aldrich), 100 U/ml penicillin and 100 μg/ml streptomycin (Sigma-Aldrich) and used for the generation of lentivirus. Sf9 insect cell line (CVCL_0549) was used to produce high titer of baculovirus stocks cultured in Sf-900 II medium (Life Technologies) supplemented with 10% (v/v) heat-inactivated FBS, (fetal bovine serum Sigma-Aldrich), 100 U/ml penicillin, 100 μg/ml streptomycin (Sigma-Aldrich) and 1% (v/v) L-glutamine (Life Technologies). Hi5 insect cell line (CVCL_C190) was used for KIR2DL-Fc fusion protein production and cultured in Express Five serum free medium (Life Technologies) supplemented with 1% (v/v) L-glutamine (Life Technologies). The HLA class I deficient B cell line 721.221 (RRID: CVCL_6263) (38) was used for expressing HLA-C1 and -C2 allotypes (**Table 1**). For this, the extracellular domains of HLA-C allotypes were obtained from GeneSynthesis (Thermo Fisher) and cloned into a lentiviral transfer vector (pSIP-ZsGreen) with a puromycin resistance. For the production of lentivirus, Lipofectamine 3000 (Life Technology) was used to transfect HEK293T/17 cells with the lentiviral transfer vector with the gene of interest, a VSV-G envelope vector (pHEF-VSVF; NIH HIV Reagent Program) and a HIV-1 Gag-Pol packaging vector (psPAX2; NIH HIV Reagent Program). After 48 h, the supernatant containing the lentivirus was harvested and used for the transduction of the 721.221 cells. 3 days post-transduction, HLA-C^+^ 721.221 cells were selected with 1 μg/ml puromycin (Sigma-Aldrich) and later sorted for high HLA-C expression by fluorescence-activated cell sorting. HLA-C-721.221 cell lines were cultured in complete RPMI-1640 medium (Life Technology) supplemented with 10% (v/v) heat-inactivated FBS (Sigma-Aldrich), 100 U/ml penicillin, 100 μg/ml streptomycin (Sigma-Aldrich) and 1 μg/ml puromcyin at 37°C and 5% CO_2_.

**Table 1:** Overview of generated HLA-C expressing 721.221 cell lines.

Cell line	Allotypes
**HLA-C1-721.221 cell lines**	*01:02, *03:03, *03:04, *07:01, *07:02, *12:03, *14:02, *16:01
**HLA-C2-721.221 cell lines**	*02:02, *04:01, *05:01, *06:02

### Generation of KIR2DL-Fc Fusion Proteins

KIR2DL1*001-Fc, KIR2DL2*003-Fc and KIR2DL3*001-Fc fusion constructs were produced as described in Hilton et al. (39). Further KIR2DL-Fc fusion constructs (**Table 2**) were generated by site-directed mutagenesis (Agilent Technologies). KIR2DL-Fc fusion constructs were co-transfected with linearized baculovirus (Expression Systems) into Sf9 insect cells with Cellfectin II (Invitrogen). Sf9 cells were cultured for 7 days in Sf-900 II medium (Life Technologies) at 115 rpm, 27°C and 5% CO_2_. To produce high titer of baculovirus, 3 more rounds of amplification were performed by culturing Sf9 cells in Sf-900 II medium supplemented with 10% (v/v) heat-inactivated FBS (Sigma-Aldrich), 100 U/ml penicillin, 100 μg/ml streptomycin (Sigma-Aldrich) and 1% (v/v) L-glutamine (Life Technologies) for 4-5 days with the respective virus stock. Hi5 insect cells were cultured in Express Five serum free medium (Life Technologies) supplemented with 1% (v/v) L-glutamine (Life Technologies). KIR2DL-Fc fusion proteins were produced by infecting Hi5 insect cells with P3 viral stock for 72 h at 115 rpm, 27°C and 5% CO_2_. The supernatant was collected by centrifugation and filtration. To isolate the KIR2DL-Fc fusion proteins, the supernatant was neutralized with HEPES buffer (Thermo Fisher Scientific) and incubated with protein A Sepharose beads (Thermo Fisher Scientific) rotating overnight. The KIR2DL-Fc fusion proteins were washed with PBS and eluted with 100 mM glycine (pH 2.7) and immediately neutralized with 1 M Tris (pH 9). The eluted proteins were desalted by using a Sephadex G-25 desalting column (GE Healthycare). Protein concentration was measured with a BCA protein assay (Thermo Fisher Scientific).

**Table 2:** Overview of generated KIR2DL-Fc fusion proteins.

	Allotypes
**KIR2DL1-Fc fusion proteins**	*001, *003, *004, *020, *022
**KIR2DL2-Fc fusion proteins**	*001, *003, *009
**KIR2DL3-Fc fusion proteins**	*001, *002, *009, *016

The symbol "*" represents a separator between the gene and the allele group. In this instance the allele group of the respective KIR2DL1, L2 and L3 proteins.

### KIR2DL-Fc Binding Assay

HLA-C-721.221 cell lines and untransduced 721.221 were incubated with 25 μg/ml KIR2DL-Fc fusion protein for 15 min at 4°C and then washed and stained with a LIVE/DEAD fixable Near-IR Dead Cell staining kit (Invitrogen) and with a secondary F(ab)2 goat anti-human IgG-PE antibody (Invitrogen). After additional washing steps, the cells were fixed in 1x CellFIX (BD Bioscience) and the binding of the KIR2DL-Fc fusion protein was analyzed *via* flow cytometry. As a negative control, cells were only stained with anti-human IgG-PE without any KIR2DL-Fc fusion protein. HLA-C expression of all transfected and untransfected 721.221 cell lines was assessed by flow cytometry using the HLA-ABC antibody W6/32 (**Supplementary Figure 1**). Binding of KIR2DL-Fc fusion proteins was normalized to the negative control and adjusted for the HLA-ABC expression of the respective 721.221 cell lines.

### Phenotypical Characterization of PBMCs From Healthy and HIV-1-Infected Individuals

PBMCs from healthy and untreated HIV-1-infected individuals were gently thawed by adding dropwise complete medium supplemented with 25 U/ml Benzonase Nuclease (Merck Milipore Novagen) followed by a 30 min incubation at 37°C and 5% CO_2_. After counting and washing with PBS, cells were incubated with LIVE/DEAD fixable Near-IR Dead Cell staining kit (Invitrogen) and the following antibodies at 4°C: anti-CD3-PerCP-Cy5.5 (clone UCHT1, BioLegend), anti-CD4-BV650 (clone RPA-T4, BioLegend), anti-CD8-AF700 (clone EB6B, BioLegend), anti-CD14-APC-Cy7 (clone HCD14, BioLegend), anti-CD19-APC-Cy7 (clone HIB19, BioLegend), anti-CD56-BUV395 (clone NCAM16.2, BD Optibuild), anti-CD16-BV785 (clone 3G8, BioLegend), anti-CD57-BV510 (clone QA17A04, BioLegend), anti-NKG2A-PE-Vio615 (clone REA110, Miltenyi), anti-NKG2C-BUV563 (clone 134591, BD Optibuild), anti-KIR2DL1/S1-APC (clone EB6B, Beckman Coulter), anti-KIR2DL1/S5-PE (clone 134211, R&D Systems), anti-KIR2DL2/L3/S2-BV711 (clone DX27, BD Optibuild), anti-KIR2DL3-AF488 (clone 180701, R&D Systems), anti-KIR3DL1-AF700 (clone DX9, BioLegend), anti-KIR3DL1/L2-PE-Vio770 (clone 5.133, Miltenyi) and anti-KIR2DS4-Biotin (clone JJC11.6, Miltenyi) with secondary Strepdavidin-BV421 (BioLegend). Cells were washed and then fixed with FluoroFix Buffer (BioLegend). Cells were analyzed by flow cytometry.

### Viral RNA Isolation, cDNA Synthesis and Genomic DNA Isolation

Viral RNA from plasma samples of HIV-1-infected individuals was isolated using the High Pure Viral RNA Kit (Roche) and then used for a reverse transcription reaction (SuperScript III One-Step RT-PCR, Invitrogen) with specific Vpu outer-revers primer (sense primer: 5’-CCT AGA CTA GAG CCC TGG AAG CAT-3’, anti-sense primer: 5’-TTC TTG TGG GTT GGG GTC TGT-3’) described by Pickering et al., 2014 (38). Genomic DNA from PBMCs was isolated using the DNeasy Blood & Tissue Kit (QIAGEN).

### Vpu Sequencing

cDNA generated from viral RNA and genomic DNA isolated from PBMC of HIV-1-infected individuals was amplified and prepared for Vpu sequencing *via* PCR (Platinum SuperFi DNA Polymerase, Invitrogen) using gene specific Vpu inner-primers (38) and overhangs of Illumina-sequencing compatible adapter sequences (highlighted in bold) (sense primer: 5’-**TCG TCG GCA GCG TCA GAT GTG TAT AAG AGA CAG** TAA TAC GAC TCA CTA TAG GCA GGA AGA AGC GGA GAC A-3’, anti-sense primer: 5´-**GTC TCG TGG GCT CGG AGA TGT GTA TAA GAG ACA G**CA GGA AAC AGC TAT GAC CCC ATA ATA GAC TGT GAC-3´). In a second PCR using the KAPA HiFi HotStart ReadyMix PCR Kit (Roche) the Illumina-sequencing compatible adapter overhangs served as a template to add dual indices (Nextera XT Index Kit, Illumina) for multiplexing and to complete the Illumina sequencing adapters. Prior to sequencing samples were pooled according to their concentration measured *via* Qubit dsDNA High Sensitivity (Invitrogen). The amplicon size of each pool was assessed on a TapeStation 4200 High Sensitivity D1000 Screen Tape (Agilent Technologies) to dilute the samples to a concentration of 2 nM. Paired-end sequencing of the generated libraries was performed on an Illumina MiSeq platform (2x250 bp) aiming for 0.1 - 0.2 million reads per sample.

### Vpu Sequence Analysis

Raw paired-end reads were aligned to indexed reference sequence AF324493.2 from NCBI GenBank using bwa mem command (40). Resulting SAM alignment files were converted to BAM files, sorted and indexed using samtools (41). Variants were detected using GATK HaplotypeCaller (42) using “–dont-use-soft-clipped-bases true” option, and to identify variant with maximum frequency “–max-alternate-alleles 1” option was used. In downstream process low quality variants were filtered out using following parameters from GATK Best Practices recommendations: “DP < 25 || QD < 2.0 || MQ < 30.0 || FS > 60.0 || MQRankSum < -12.5 || ReadPosRankSum < -8.0” (43). Filtered high quality variants meeting the criteria were then taken as input to create mutation VPU nucleotide sequence taken 6061:6306 as reference coordinates using GATK FastaAlternateReferenceMaker command (44).

### HLA Class I and KIR Genotyping

Genomic DNA was isolated from cryopreserved PBMCs with the DNeasy Blood and Tissue Kit (QIAGEN). HLA class I and KIR genotyping was performed by the DKMS Life Science Lab, Dresden, Germany as described in (45, 46).

### Data Analysis and Statistics

The acquisition of flow cytometric data was performed with a BD LSRFortessa (BD Bioscience) in the core facility Fluorescence Cytometry at the Leibniz Institute of Virology and analyzed using FlowJo software 10.7.1 (BD Life Sciences). Graphical and statistical analyses were performed using GraphPad Prism 9.0.1 software (GraphPad Software, La Jolla, CA, USA). Multiple linear regression analysis was performed to assess the impact of HIV-1 status, age and sex on the expression of NK cell receptors (**Figure 2C**). One-way ANOVA, test for linear trend was used to test for gene-dose effects (Figure 2A, B, D, E). A non-parametric statistical test (Mann-Whitney) was applied to test for differences between two groups. Adjustment for multiplicity was applied to comparisons of interest using false discovery rate (FDR) with Q = 5% (Benjamini/Krieger/Yekutieli). Bonferroni correction was used in one instance (**Figures 3A, B**). Variation in the frequency of amino acid residues between individuals of different HLA-C genotypes was analyzed by chi square tests. Statistical parameters are stated in the results section as well as in the figure legends.

## Results

### KIR2DL/HLA-C Allotype Combinations Display Differential Binding Affinities

**Figure 1:**
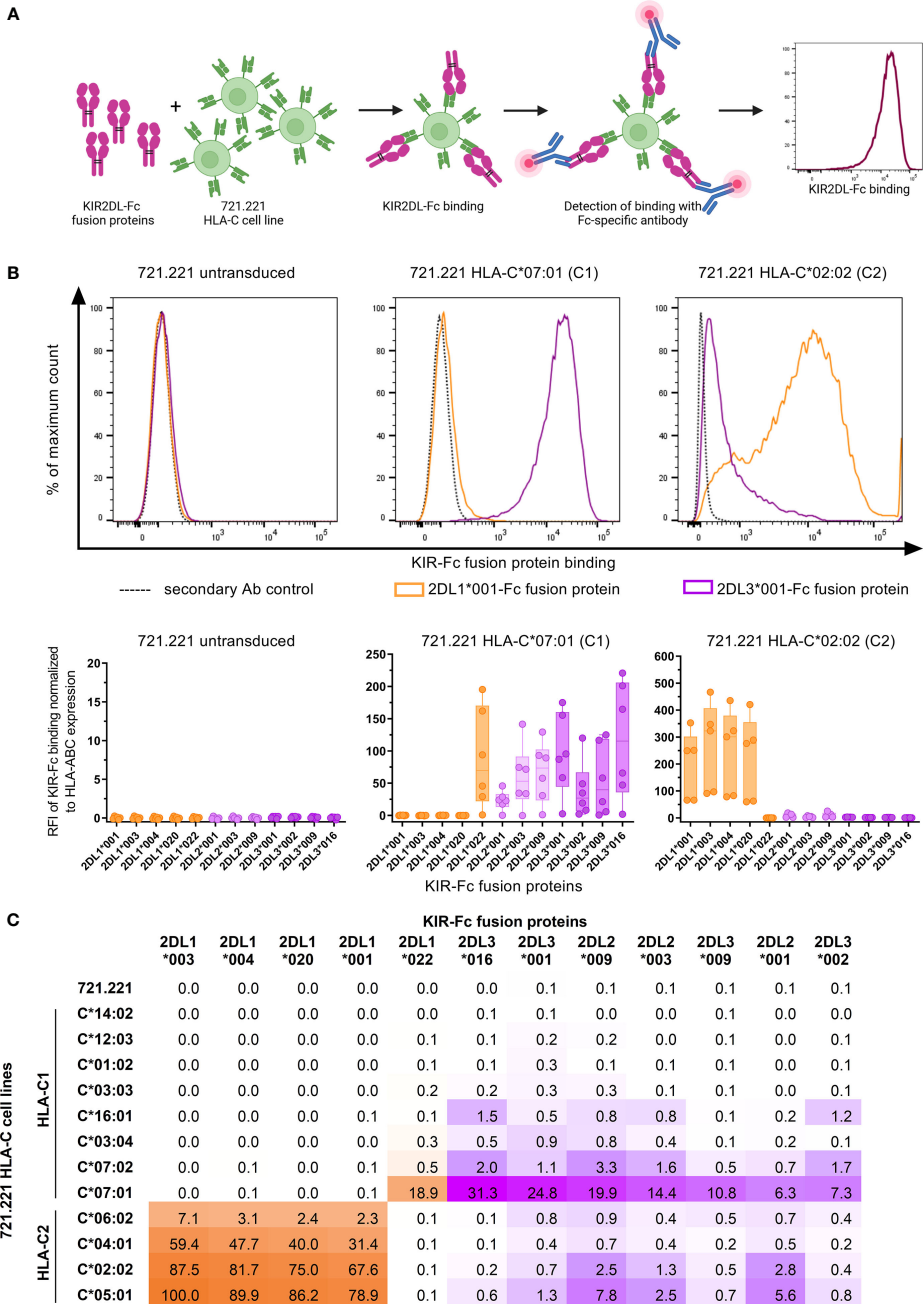
KIR2DL-Fc fusion proteins display differential binding affinities to HLA-C expressing 721.221 cell lines. **(A)** Overview of the assessment of binding affinities between HLA-C expressing 721.221 cell lines and KIR2DL-Fc fusion proteins using flow cytometry. Created with BioRender.com. **(B)** Upper panel: Representative histograms depicting binding of KIR2DL1*001-Fc (orange), KIR2DL3*001-Fc (purple) and the respective secondary antibody (Ab) control (black, dotted) to 721.221 (.221),.221-C*07:01 (C1) and .221-C*02:02 (C2) cell lines. Expression was quantified as fluorescence intensity (x-axis). Lower panel: Cumulative data showing binding of KIR2DL-Fc fusion proteins to .221, .221-C*07:01 (C1) and .221-C*02:02 (C2) cell lines. Binding of KIR2DL1-Fc (orange), KIR2DL2-Fc (light purple) and KIR2DL3-Fc (dark purple) allotypes is displayed as relative fluorescence intensity (RFI) normalized to the secondary antibody only control and adjusted for HLA-ABC expression of the respective 721.221 cell lines. Box plots show median and 25%/75% percentile. Data points represent at least five technical replicates (n ≤ 6). **(C)** Summarizing results of binding assays between 12 KIR2DL-Fc allotypes and .221s expressing 12 different HLA-C allotypes. Highest binding value was set to 100% and all other values were calculated in relation to the 100% value.

Previous assessments of KIR binding affinities to HLA class I have repeatedly confirmed the overall specificities of KIRs to different groups of HLA class I molecules (7, 10, 12). Further stratification of HLA class I and KIR molecules into specific allotypes revealed that binding affinities are exhibited as a continuum, ranging from strong affinities to no binding at all (8, 47, 48). In order to assess the specificity and affinity of the receptors KIR2DL1, KIR2DL2, and KIR2DL3 (henceforth KIR2DL) to various HLA-C allotypes in a cellular system, we assessed binding of various KIR2DL-Fc fusion proteins to HLA-C expressing cell lines using flow cytometry (**Figure 1A**). Representative and cumulative data in **Figure 1B** display the overall binding specificities of the three KIR2DL types. All generated KIR2DL-Fc constructs did not bind to untransduced 721.221, while KIR2DL1-Fc allotypes were predominantly associated with binding of HLA-C group 2 (HLA-C2), but did not recognize HLA-C group 1 (HLA-C1) on 721.221s with the exception of KIR2DL1*022. KIR2DL1*022 is characterized by an amino acid substitution at position 44 which leads to a switch in its binding specificity towards HLA-C1 ligands (49). Conversely, KIR2DL2-Fc and KIR2DL3-Fc displayed binding affinities to both HLA-C1 and -C2. For comparison of the binding affinities of all tested KIR2DL/HLA-C allotype combinations, the highest value for KIR2DL-Fc binding was set to 100% and all other values were calculated accordingly (**Figure 1C**). The displayed affinity matrix indicated hierarchies that applied to KIR2DL1 as well as HLA-C2 allotypes. E.g. 2DL1*003 showed the highest affinities to HLA-C2 cell lines of all tested KIR2DL1 allotypes independent of the HLA-C allotype follwes by 2DL1*004, 2DL1*020 and 2DL1*001. In turn, highest KIR2DL1 binding was observed for the HLA-C2 cell line expressing C*05:01 irrespective of the KIR2DL1 allotype. This was followed by C*02:02, C*04:01 and C*06:02. Overall KIR2DL2-Fc and KIR2DL3-Fc constructs displayed a lower binding affinity to HLA-C expressing cell lines in general and showed a more ambiguous affinity pattern. E.g. the KIR2DL3*016/C*07:01 combination showed the strongest binding of all KIR2DL2/L3 allotypes to HLA-C1 cell lines. But for HLA-C2 cell lines KIR2DL2*009 showed the highest affinity, i.e. C*05:01. Of note, the HLA-C1 cell lines C*01:02, C*03:03, C*12:03 and C*14:02 did not allow for any KIR2DL-Fc binding in our experimental setting, whereas C*03:04, C*07:02 and C*16:01 demonstrated only low binding affinities to the KIR2DL2/L3-Fc fusion proteins. C*07:01 allowed for stronger binding of KIR2DL2/L3 allotypes than any other tested C1 cell line. Altogether, the performed KIR2DL-Fc fusion protein assays showed high binding affinities of KIR2DL1/HLA-C2 combinations but no binding of KIR2DL1 to HLA-C1. Furthermore, KIR2DL2/L3-Fc fusion proteins had only low binding affinities for both, HLA-C1 and -C2 expressing 721.221 cell lines.

### HIV-1 Infection Is Associated With an Altered NK Cell Receptor Repertoire

**Figure 2:**
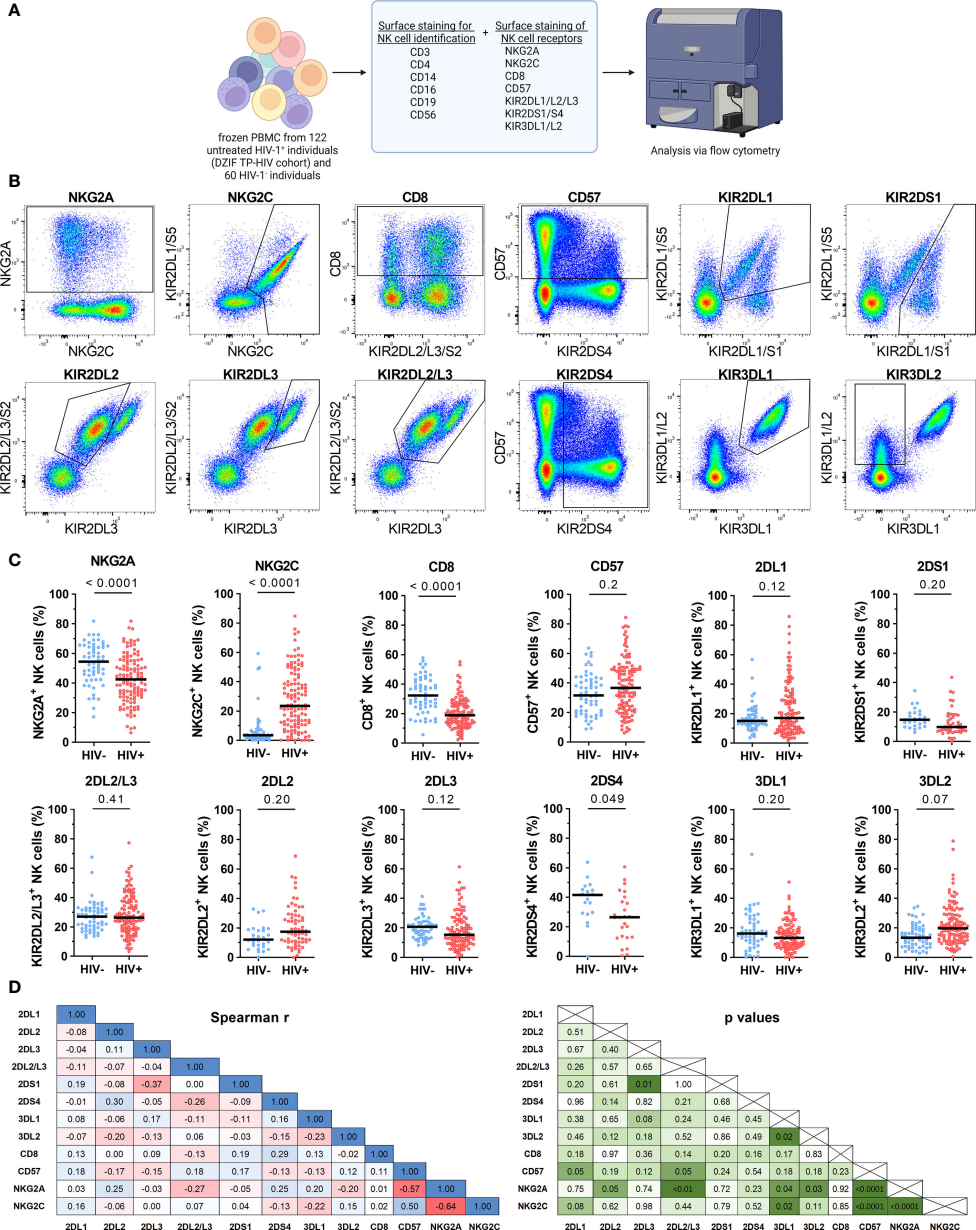
Cell surface expression of NK cell receptors of HIV-1^-^ and untreated HIV-1^+^ individuals. **(A)** Overview of the flow cytometric assessment of NK cell receptor expression on NK cells from HIV-1^-^ (n = 60) and untreated HIV-1^+^ (n = 122) individuals. Created with BioRender.com **(B)** Respective expression patterns of the NK cell receptors NKG2A, NKG2C, CD8, CD57, KIR2DL1, KIR2DS1, KIR2DL2, KIR2DL3, KIR2DL2/L3, KIR2DS4, KIR3DL1 and KIR3DL2 on bulk NK cells as well as the respective gating. **(C)** Scatter plots displaying the percentage of NKG2A^+^, NKG2C^+^, CD8^+^, CD57^+^, KIR2DL1^+^, KIR2DS1^+^, KIR2DL2/L3^+^, KIR2DL2^+^, KIR2DL3^+^, KIR2DS4^+^, KIR3DL1^+^ and KIR3DL2^+^ cells within bulk NK cells in HIV-1^-^ (blue) and HIV-1^+^ (red) individuals. Each data point represents one donor. Donors lacking the respective gene, containing a gene deletion (KIR2DS4-del) or a null allele (KIR3DL1*004) were excluded (NKG2A/NKG2C/CD8/CD57/KIR2DL2/L3: HIV-1^-^: n = 60, HIV-1^+^: n = 122; KIR2DL1: n = 59, n = 118; KIR2DS1: n = 26, n = 47; KIR2DL2: n = 28, n = 64; KIR2DL3: n = 55, n = 111; KIR2DS4: n = 19, n = 25; KIR3DL1: n = 55, n = 108). **(D)** Correlation analyses between the frequencies of all tested receptor^+^ NK cell subsets from HIV-1^+^ individuals. Left panel: r_s_ values; right panel: p values. **Data information: (C)** Bars indicate the median for each group. Multiple linear regression analysis was used to determine differences between HIV-1^-^ and HIV-1^+^ individuals. **(D)** Spearman rank analysis. **(C, D)** p values were adjusted for multiple comparisons (Benjamini/Krieger/Yekutieli).

Next, we investigated the impact of HIV-1 infection on the expression patterns of NK cell receptors. For this, we assessed the expression of eleven NK cell receptors within 60 HIV-1^-^ and 122 untreated HIV-1^+^ individuals (**Table 3**; **Figure 2A**). Exemplary expression patterns for all analyzed NK cell receptors and the applied gating are shown in **Figure 2B**. Initial comparative analyses of bulk NK cells, including adjustment for age and sex, suggested significant changes in the receptor repertoire between HIV-1^+^ individuals and the HIV-1^-^ control group (**Figure 2C** and **Supplementary Table 1**). We observed a lower percentage of NK cells expressing the inhibitory receptor NKG2A in HIV-1^+^ individuals (median: 42.5%) compared to our control group (54.4%, p < 0.0001 *vs*. HIV-1-), whereas the percentage of NK cells expressing the activating counterpart NKG2C was considerably increased (23.5% *vs*. 3.5%, p < 0.0001 *vs*. HIV-1-). The relative frequency of NK cells expressing CD57, a marker for terminal differentiation, was slightly higher in HIV-1^+^ individuals (36.7%) compared to the HIV-1^-^ control group (31.5%) but did not reach statistical significance after adjustment for age and sex (p = 0.2). Percentage of CD57^+^ NK cells was positively associated with the percentage of NKG2C^+^ NK cells (r_s_ = 0.5, p < 0.0001) and negatively with the percentage of NKG2A^+^ NK cells (r_s_ = -0.57, p < 0.0001; **Figure 2D**). The relative frequency of CD8^+^ NK cells was reduced in HIV-1^+^ individuals (18.9%) in comparison to HIV-1^-^ donors (32.3%, p < 0.0001) but did not correlate with any other investigated NK cell receptor. For most KIRs expression patterns seemed to be unaffected by HIV-1 infection. No differences in the relative frequency of KIR2DL1^+^ cells (p = 0.12) and KIR2DL2/L3^+^ cells (p = 0.41) were observed, even after stratification into KIR2DL2 (p = 0.2) and KIR2DL3 (p = 0.12). The activating KIRs, KIR2DS1 (p = 0.2) remained unaltered as well but KIR2DS4 showed lower relative frequencies in HIV-1^+^ individuals (26.5% *vs*. 41.9%, p = 0.049). The proportion of KIR3DL1^+^ NK cells was not significantly altered (p = 0.20), even when adjusted for its ligand Bw4 or stratified by KIR3DL1 allotype groups (**Supplementary Figure 2**) in contrast to KIR3DL2, which exhibited increased relative frequencies in HIV-1^+^ individuals (19.7% *vs*. 13.4%, p = 0.07). Analysis of relative frequencies and absolute cell numbers of the respective receptor^+^ NK cells revealed a significant positive correlation for all receptors (**Supplementary Figure 3**), indicating that the observed changes represent an actual expansion or contraction of NK cell subsets.

**Table 3:** Demographic and clinical profile of HIV-1^-^ and HIV-1^+^ individuals.

		HIV-1^-^ individuals	HIV-1^+^ individuals
	Number total	60	122
**Demographic** **data**	**Age in years** Median (Min; Max)	N = 43 30 (22; 64)	N = 122 36.5 (20; 73)
	**Sex** Male, number (%) Female, number (%)	N = 44 21 (47.7) 23 (52.3)	N = 122 114 (93.4) 8 (6.6)
**Clinical data**	**Viral load in copies/ml** Median (Min; Max)	n.d	N = 121 516,000 (40; 170,000,000)
	**CD4 T cells in cells/μL** Median (Min; Max)	n.d	n=118 438 (22; 2,601)
	**CD4 T cells in %** Median (Min; Max)	n.d	n=116 24.6 (3; 54)
	**CD8 T cells in cells/μL** Median (Min; Max)	n.d	N = 84 936 (177; 6,636)
	**CD8 T cells in %** Median (Min; Max)	n.d	N = 79 50 (29; 85.9)
	**CD4/CD8 ratio** Median (Min; Max)	n.d	N = 84 0.49 (0.1; 1.86)
	**NK acells in cells/μL** Median (Min; Max)	n.d	N = 118 164 (14; 2,004)

Subsequently, we investigated whether the altered NK cell receptor repertoire was attributed to changes of the distribution of the three major CD56 NK cell subsets. Based on the expression levels of CD56, NK cells can be distinguished into CD56^Bright^ (CD16^-^/dim), CD56^Dim^ (CD16^+^) and CD56^Negative^ sub-populations (CD16^+^), each displaying specific receptor profiles on their own (50). Compared to the HIV-1- control group, the frequency of CD56^Bright^ NK cells was significantly decreased within the NK cell compartment of HIV-1- infected individuals (p < 0.0001) (**Supplementary Figure 4**). Conversely, the proportion of CD56^Negative^ NK cells was increased (p < 0.0001). Subsequently, the three CD56 NK cell subsets were analyzed for the expression of the eleven NK cell receptors (**Table 4**). Changes of NK cell receptor profiles were observed in all CD56 NK cell subsets. In the CD56^Bright^ NK cell subset, eight of the investigated NK cell receptors, namely KIR2DL1, KIR2DL2, KIR2DL3, KIR2DS4, KIR2DS1, KIR3DL1, CD57 and NKG2C exhibited an increased frequency in HIV-1^+^ individuals, although it should be noted that percentage point differences were rather marginal for all tested KIRS. In contrast, NKG2A and CD8 were significantly decreased. In the CD56^Dim^ subset, CD8^+^ NK cells showed decreased frequencies, while the KIR3DL2^+^ NK cell subset was enlarged. CD56^Negative^ NK cells showed similar changes in the expression patterns as the CD56^Dim^ subset in both HIV-1^-^ and HIV-1^+^ groups, although CD56^Negative^ NK cells displayed an overall lower percentage of receptor^+^ cells compared to CD56^Dim^ NK cells. Taken together, we observed reduced percentages of CD8^+^ NK cells in all CD56 NK cell subsets of HIV-1^+^ individuals compared to HIV-1^-^ individuals, whereas the NKG2C^+^ subset was enlarged. In contrast, changes in the KIR profiles between HIV-1^+^ and HIV-1^-^ individuals were rather marginal in the CD56^Dim^ subset in which KIRs are predominantly expressed, with the exception of KIR3DL2.

**Table 4:** NK cell receptor expression in NK cell subsets.

			CD56^Bright^			CD56^Dim^			CD56^Negative^		
	HIV-	HIV+	HIV-	HIV+		HIV-	HIV+		HIV-	HIV+	
receptor	n	n	median(25%/75% percentile)	median(25%/75% percentile)	p value*	median(25%/75% percentile)	median(25%/75% percentile)	p value*	median(25%/75% percentile)	median(25%/75% percentile)	p value*
NKG2A	60	122	95.9 (94.1/97.1)	93.7 (89.7/96.9)	0.0003	52.5 (41.3/63.2)	41.4 (29.6/54.7)	0.002	32.9 (23.4/45.3)	29.9 (20.6/42.7)	0.1
NKG2C	60	122	10.2 (7.1/15.9)	14.0 (8.0/20.1)	0.04	2.6 (1.7/5.4)	24.9 (11.0/42.0)	<0.0001	1.8 (1.0/3.4)	17.1 (6.9/33.9)	<0.0001
CD8	60	122	23.8 (18.7/31.1)	17.2 (11.0/23.3)	0.003	37.1 (29.4/45.8)	19.5 (13.3/28.0)	<0.0001	28.6 (20.7/33.1)	14.3 (8.4/19.2)	<0.0001
CD57	60	122	0.5 (0.2/1.0)	0.8 (0.4/2.2)	0.0001	34.2 (23.4/46.2)	40.2 (26.0/52.9)	0.13	9.5 (6.4/13.8)	14.0 (7.5/27.1)	0.052
KIR2DL1	59	118	1.4 (0.8/1.9)	2.2 (1.2/4.0)	0.0003	17.9 (14.2/23.6)	18.9 (9.9/36.8)	0.36	10.4 (7.7/13.1)	9.3 (4.4/17.7)	0.45
KIR2DS1	26	47	0.9 (0.6/1.4)	1.5 (0.8/2.4)	0.006	16.5 (11.2/20.5)	9.8 (6.8/18.5)	0.052	11.6 (7.7/17.5)	8.2 (4.7/12.6)	0.056
KIR2DL2/L3	60	122	1.9 (1.5/2.9)	3.2 (1.8/6.1)	0.0001	29.7 (21.2/33.8)	27.2 (20.0/39.0)	0.49	18.6 (12.6/24.0)	19.6 (12.3/28.1)	0.13
KIR2DL2	28	64	1.1 (0.6/1.9)	2.0 (1.1/3.4)	0.035	12.3 (8.3/19.6)	17.2 (10.8/29.8)	0.77	10.8 (5.8/15.8)	13.2 (7.6/22.3)	0.45
KIR2DL3	55	111	1.4 (0.8/1.9)	2.0 (1.1/3.5)	0.04	21.7 (14.4/25.5)	16.4 (10.1/26.3)	0.074	12.6 (8.8/17.6)	10.1 (6.1/18.9)	0.40
KIR2DS4	19	25	3.4 (2.1/4.3)	4.2 (1.9/8.8)	0.042	45.4 (30.5/49.7)	27.3 (10.3/40.3)	0.27	30.4 (20.1/37.9)	23.1 (8.2/29.8)	0.71
KIR3DL1	55	108	1.2 (0.6/2.0)	2.8 (1.5/4.3)	0.003	15.5 (9.0/24.0)	12.8 (8.0/20.4)	0.23	8.0 (4.7/12.6)	8.6 (5.1/14.1)	0.42
KIR3DL2	60	122	11.3 (7.3/15.0)	10.5 (6.9/13.8)	0.21	14.9 (9.0/22.9)	20.4 (13.2/29.2)	0.048	10.4 (6.4/16.6)	17.6 (10.5/27.1)	0.0003

*Multiple linear regression analysis (HIV, sex, age). p values were adjusted for multiple comparison (Benjamini/Krieger/Yekutieli).

### *HLA-C* and *KIR2DL* Genotypes Impact Frequency of KIR2DL^+^ NK Cells

**Figure 3:**
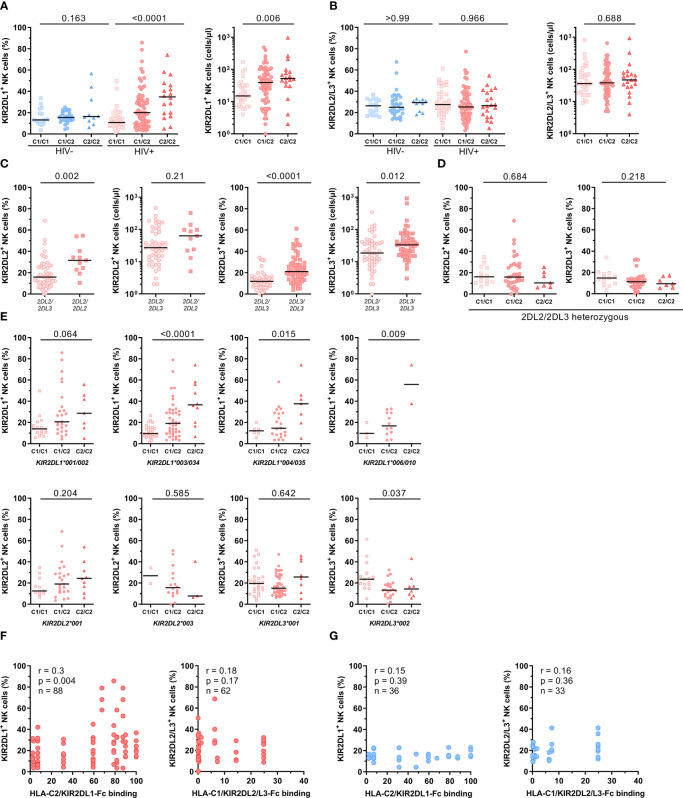
Impact of *HLA-C* and *KIR* genotypes on the frequency of NK cells expressing HLA-C-binding KIRs. **(A)** Percentage of KIR2DL1^+^ cells within bulk NK cells in HIV-1^-^ and HIV-1^+^ individuals (left panel). Absolute numbers of KIR2DL1^+^ NK cells in HIV-1^+^ individuals (right panel). **(B)** Percentage of KIR2DL2/L3^+^ cells within bulk NK cells in HIV-1^-^ and HIV-1^+^ individuals (left panel). Absolute numbers of KIR2DL2/L3^+^ NK cells in HIV-1^+^ individuals (right panel). (A/B) Donors were stratified by *HLA-C* group genotypes. Genotypes were defined by the presence of HLA-C allotypes carrying either a C1 or C2 epitope (C1/C1 = C1^homozygous^, C1/C1 = C1/C2^heterozygous^, C2/C2 = C2^homozygous^). **(C)** Percentage and absolute cell numbers of KIR2DL2^+^ NK cells and KIR2DL3^+^ NK cells in HIV-1^+^ individuals stratified by the presence of *KIR2DL2* or *KIR2DL3* alleles. **(D)** Percentage of KIR2DL2^+^ and KIR2DL3^+^ NK cells in *KIR2DL2/L3* ^heterozygous^ HIV-1^+^ individuals stratified by *HLA-C* group genotypes. **(E)** Percentage of KIR2DL1^+^, KIR2DL2^+^ and KIR2DL3^+^ NK cells in HIV-1^+^ individuals stratified by *HLA-C* group genotypes and *KIR2DL* alleles. **(F)** Correlation between percentage of KIR2DL1^+^ NK cells and HLA-C2/KIR2DL1-Fc binding, KIR2DL2/L3^+^ NK cells and HLA-C1/KIR2DL2/L3-Fc binding in HIV-1^+^ individuals (red) stratified by KIR/HLA binding affinities determined in **Figure 1**. **(G)** Correlation between percentage of KIR2DL1^+^ NK cells and HLA-C2/KIR2DL1-Fc binding, KIR2DL2/L3^+^ NK cells and HLA-C1/KIR2DL2/L3-Fc binding in HIV-1^-^ individuals (blue) stratified by KIR/HLA binding affinities determined in **Figure 1** **. Data information:** Black bars display the median. **(A, B, D, E)** One-way ANOVA, test for linear trend. **(C)** Mann-Whitney test. P values were adjusted for multiple comparisons (**A, B, D:** Bonferroni; **E**: Benjamini/Krieger/Yekutieli). **(F, G)** Spearman rank correlation.

To investigate the influence of *KIR2DL/HLA-C* genotype combinations on KIR2DL expression on NK cells, HIV-1^+^ and HIV-1^-^ individuals were stratified according to their *HLA-C* allotypes. HIV-1^+^ individuals showed significant differences in the frequency of KIR2DL1^+^ NK cells between the generated groups, while no differences were detected in the HIV-1^-^ control group **(** **Figure 3A** **)**. HIV-1^+^ individuals carrying the cognate HLA-C2 ligand (C1/C2, C2/C2) showed an increased frequency of KIR2DL1^+^ NK cells, whereas individuals without *HLA-C2* alleles (C1/C1) exhibited a generally lower percentage of KIR2DL1^+^ NK cells (left panel, linear trend: p < 0.0001). This effect was not due to a skewed distribution of KIR2DL1-C^245^ and KIR2DL1-R^245^ allotypes between HIV-1^+^ and HIV-1^-^ individuals or between *HLA-C2* ^+^ or *HLA-C2* ^-^ donors (data not shown), which was previously shown to affect KIR2DL1 expression (51). The observed gene-dose dependent effect of *HLA-C2* alleles within the HIV-1^+^ group was also observed when using absolute cell numbers as a readout (right panel, p = 0.006). In contrast, presence of the HLA-C1 ligand did not affect the proportion or cell number of NK cells expressing the respective receptors KIR2DL2/L3 in either HIV-1^+^ or HIV-1^-^ individuals (**Figure 3B**, percentage HIV-1^-^: p > 0. 99, HIV-1^+^: p = 0.97, cell number: p = 0.69).

Given that potential changes of KIR2DL2/L3 frequencies between HIV-1^+^ and HIV-1^-^ groups were masked by the previously observed opposing directions of KIR2DL2^+^ and KIR2DL3^+^ NK cell frequencies, we stratified KIR2DL2/3 frequencies based on their *KIR2DL2/L3* genotype (**Figure 3C**). NK cells from homozygous *KIR2DL2* individuals (KIR*2DL2/L2*) showed a significantly increased frequency of KIR2DL2^+^ NK cells compared to heterozygous (*KIR2DL2/L3*) individuals independent of the underlying *HLA-C* genotype (p = 0.002). This effect was also present in KIR2DL3^+^ NK cells when comparing *KIR2DL3* homozygous (*KIR2DL3/L3*) and heterozygous (*KIR2DL2/L3*) individuals (p < 0.0001). To exclude the skewing effect of *KIR2DL2* and *KIR2DL3* homozygosity, stratification of *HLA-C* allotypes was performed only for *KIR2DL2/L3* heterozygous HIV-1^+^ individuals (**Figure 3D**). Increased frequencies of KIR2DL2^+^ and KIR2DL3^+^ NK cells in donors with two *HLA-C1* alleles (C1/C1) were observed compared to individuals lacking *HLA-C1* alleles (C2/C2), however the overall trend did not reach statistical significance (KIR2DL2^+^: p = 0.68, KIR2DL3^+^: p = 0.22).

Based on the observed effects of homo- or heterozygosity of the *KIR2DL/L3* gene, we sought to further stratify donors based on the specific *KIR2DL* alleles (**Figure 3E**). In line with the results for KIR2DL1^+^ NK cells from all HIV-1^+^ individuals stratified by their *HLA-C* allotypes, HIV-1^+^ individuals with KIR2DL1*001/002, *003/*034, *004/*035 or *006/010 alleles showed the same distribution of KIR2DL1^+^ NK cells with the highest frequency of KIR2DL1^+^ NK cells in *HLA-C2* homozygous individuals and the lowest frequency in *HLA-C1* homozygous donors. Moreover, individuals positive for KIR2DL2*001 and with at least one *HLA-C2* allele (C1/C2, C2/C2) showed an increased frequency of NK cells expressing KIR2DL2 compared to individuals without *HLA-C2*. In contrast, individuals carrying a KIR2DL2*003 allele showed reverse KIR2DL2 frequencies with the highest percentages for *HLA-C1/C1* homozygous and the lowest percentages for *HLA-C2/C2* homozygous individuals. Although, it should be noted that the sample size for this particular allele was rather low. Stratification of *HLA-C* allotypes for different *KIR2DL3* alleles showed a slightly increased frequency of KIR2DL3^+^ NK cells in *HLA-C2/C2* individuals positive for KIR2DL3*001, whereas individuals carrying a KIR2DL3*002 allele had an increased frequency of KIR2DL3 in the group of *HLA-C1/C1* genotypes. In addition, the observed distribution of *KIR2DL1* alleles in HLA-C genotypes was also present but less pronounced when using absolute cell numbers, whereas *KIR2DL2* and *KIR2DL3* alleles showed no differences in *HLA-C* genotypes when using absolute cell numbers a s readout (**Supplementary Figure 5**). To examine a possible relationship between *KIR/HLA-C* genotype combinations and the frequency of KIR2DL^+^ NK cells, correlation analyses between the frequencies of KIR2DL^+^ NK cells and HLA-C/KIR2DL-Fc binding affinities were performed for HIV-1^-^ and HIV-1^+^ individuals (**Figures 3F, G**). For this, we included individuals heterozygous for *C1/C2* and carrying *HLA-C/KIR* combinations with corresponding binding data. Frequency of KIR2DL1^+^ NK cells showed a significant positive correlation with the assessed binding affinities for HLA-C2/KIR2DL1 combinations (r_s_ = 0.3, p = 0.004) in HIV-1^+^ individuals. On the other hand, KIR2DL1^+^ NK cell frequency and HLA-C2/KIR2DL1 binding affinities for HIV-1^-^ individuals, as well as KIR2DL2/L3^+^ NK cell frequency and HLA-C1/KIR2DL2/L3 binding affinities for HIV-1^+^ and HIV-1^-^ individuals showed no significant correlation. Overall, these results suggest that both, the underlying HLA-C and KIR genotype of an individual has a direct impact on the expression levels of the corresponding KIR in the respective NK cell pool.

### Host Genetics Impact *Vpu* Sequence Polymorphisms in Plasma and PBMC Samples

**Figure 4:**
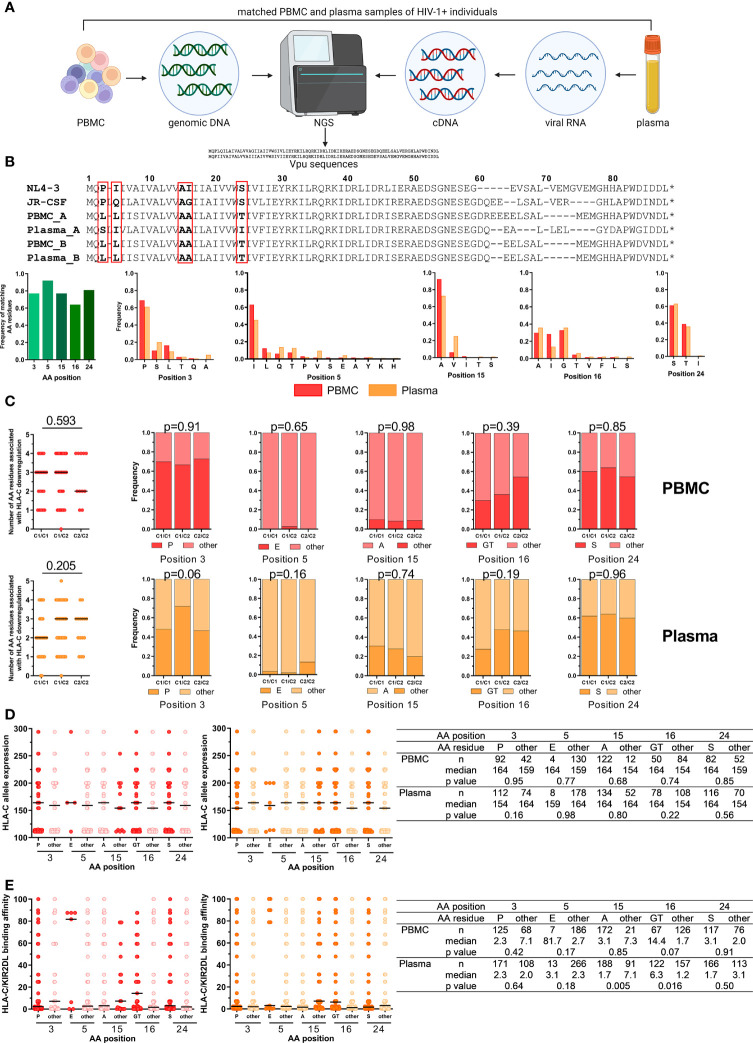
Impact of *HLA-C/KIR2DL* genotypes on Vpu sequence polymorphisms. **(A)** Overview of the work flow for Vpu sequencing from matched PBMC (n = 122) and plasma samples (n = 122) of untreated HIV-1^+^ individuals. Created with BioRender.com **(B)** Upper panel: Representative alignment of amino acid (AA) sequences of HIV-1 Vpu passing quality control. Lower panels: Frequency of matching AA residues at position 3, 5, 15, 16 and 24 between matched PBMC and plasma samples (n = 53). Frequency of individual AA residues at 3, 5, 15, 16 and 24 in PBMC (n = 67) and plasma (n = 93) samples. **(C)** Left: Dot plots displaying the cumulative number of AA residues associated with Vpu-mediated HLA-C downregulation per sequence. Right: Frequency of AA residues at position 3, 5, 15, 16 and 24. Individuals were stratified by *HLA-C* group genotypes (x-axis). Upper panel: PBMC, lower panel: Plasma. **(D)** Expression levels of HLA-C alleles of HIV-1^+^ donors. Data points are displayed for each position (3, 5, 15, 16 and 24) and AA residues. AA residues associated with HLA-C downregulation are displayed in red (PBMC, left panel) or dark orange (plasma, right panel). Summary table displaying descriptive and comparative statistics. **(E)** Binding affinities of KIR/HLA-C combinations of HIV-1^+^ donors. Data points are displayed in red (PBMC, left panel) or dark orange (plasma, right panel). Summary table displaying descriptive and comparative statistics. **Data information:** Black bars display the median. **(C)** Left panel: One-way ANOVA, test for linear trend. Right panels: Chi square test. **(D, E)** Mann-Whitney test.

Lab-adapted HIV-1 strains and primary isolates display differential abilities to modulate HLA-C expression on the surface of infected CD4^+^ T cells mediated by the accessory HIV-1 protein Vpu (29, 31). Previously, five amino acid (AA) positions have been identified at which specific residues were independently associated with HLA-C downregulation (30): Proline (P) at position 3, glutamic acid (E) at position 5, glycine (G) or threonine (T) at position 16 and serine (S) at position 24. Conversely, alanine (A) at position 15 negatively affected the ability to downregulate HLA-C. To investigate the impact of HLA-C/KIR2DL allotype combinations on Vpu sequence variations, genomic DNA and viral RNA from matching PBMC and plasma samples from 122 HIV-1^+^ individuals were isolated and sequenced with next-generation sequencing (NGS) for Vpu (**Figure 4A**). Sequences of *Vpu* from 93 plasma and 67 PBMC samples passed quality control, were translated to amino acid sequences and then aligned to the Vpu protein from the lab-adapted HIV-1 strains NL4-3 and JR-CSF (**Figure 4B**). Comparison of sequence similarity for the five analyzed AA positions between matching PBMC and plasma samples showed a frequency of matching AA residues between 0.64 and 0.92, with the lowest frequency for position 16 and the highest frequency for position 5. In addition, the frequency of all observed residues for the AA position was determined for PBMC and plasma samples. For position 3, the most common AA was proline (PBMC: 0.69, plasma: 0.61) followed by serine and leucine. Position 5 showed the highest variations in AA residues with the highest frequency for isoleucine (PBMC: 0.63, plasma: 0.45). The most common AA at position 15 was alanine (PBMC: 0.92, plasma: 0.73) followed by valine. Moreover, position 16 had comparable frequencies for alanine (PBMC: 0.29, plasma: 0.36), isoleucine (PBMC: 0.28, plasma: 0.14) and glycine (PBMC: 0.33, plasma: 0.36). Position 24 showed the lowest variations in AA residues with a serine as the most common AA (PBMC: 0.61, plasma: 0.63) followed by a threonine.

Stratification of HLA-C allotypes was performed for Vpu sequences for PBMC and plasma samples and analyzed for the five respective AA previously identified to influence HLA-C expression levels (**Figure 4C**). Vpu sequences isolated from PBMC samples from HLA-C1^+^ individuals contained a median of three of the five AA residues, whereas *HLA-C2* homozygous individuals had a median of two AA residues that were associated with HLA-C downregulation (linear trend, p = 0.593). In comparison, Vpu sequenced from viral RNA from plasma samples had three of the five AA residues in individuals with at least one *HLA-C2* allele and *HLA-C1* homozygous individuals had two (p = 0.205). However, there were no differences detectable in the frequency of AA residues associated with HLA-C downregulation when individuals were stratified by their *HLA-C* genotype.

HLA-C surface expression levels vary and correlate with the HLA-C allotype (28). To identify a potential influence of HLA-C surface expression levels on Vpu sequence variations, Vpu sequences from PBMC and plasma samples were analyzed for the five AA positions that are involved in HLA-C downregulation based on the average MFI HLA-C allele expression (**Figure 4D**). Our analysis showed no significant influence of *HLA-C* allele expression on individual AA residuces.

Finally, differences in AA residues for the five positions were analyzed based on the assessed binding affinities of HLA-C1/KIR2DL2/3 and HLA-C2/KIR2DL1 allotype combinations (**Figure 4E**). HLA-C/KIR2DL combinations with stronger binding affinities were more common in Vpu sequences from plasma samples with AA other than an alanine at position 15 (15other) (p = 0.005) or a glycine or threonine at position 16 (16GT) (p = 0.016) whereas HLA-C/KIR2DL binding affinities were not associated with specific amino acid residues in Vpu sequences from PBMC samples. Overall, these results indicated that HLA-C expression level and HLA-C/KIR2DL binding may have an influence on Vpu sequence polymorphisms.

## Discussion

In recent years multiple studies have drawn attention to the potential role of HLA-C and its cognate inhibitory receptors, KIR2DL1, KIR2DL2, and KIR2DL3, in the intrinsic control of HIV-1 infection. Most notably were two studies showing the protective effect of high HLA-C expression levels on HIV-1 progression (28) and the differential ability of HIV-1 to modulate HLA-C expression through the accessory protein Vpu (29). In contrast to Nef-mediated downregulation of HLA-A/B, the magnitude of HLA-C modulation by Vpu varies extensively across viral strains. Further investigations revealed that HIV-1 adapts its ability for downmodulation to the *HLA-C* genotype of the host (30). However, the majority of the investigated viral strains exhibited only a marginal ability to reduce HLA-C expression on infected cells (30) indicating that other factors than escape from CTL recognition through HLA-C downmodulation may contribute to Vpu sequence polymorphisms. Previous reports by our group demonstrated that KIR2DL^+^ NK cells are able to sense HIV-1-mediated alterations of HLA-C (31). Based on these studies, we hypothesized that differential binding affinities across KIR and HLA-C allotypes in part pre-determine KIR2DL^+^ NK cell induced immune pressure and Vpu-associated viral escape.

Our results demonstrated a hierarchy of affinities along the various combinations and confirmed the binding specificities of KIR2DL1 (HLA-C2), KIR2DL3 (HLA-C1) and KIR2DL2 (HLA-C1/-C2) (52). These results are largely consistent with those from the cell-free system of Hilton et al. who used KIR-Fc fusion constructs and microbeads, each coated with a different HLA class I allotype (39). While specificities between KIR2DL and HLA-C are defined by position 44 of the KIR protein (15) and position 80 of the α1 domain of HLA-C (7), other amino acid positions within both molecules fine-tune avidity (19, 53). Data on the functional consequences of the variegated affinities in the context of HIV-1 infection have been limited to KIR3DL1 (54, 55). However, multiple studies have shown that changes in the HLA class I presented peptidome can modulate binding to KIRs and subsequently impact NK cell functions (33, 56, 57). Moreover, HIV-1 sequence polymorphisms have been associated with *KIR2DL* genes alone and in combination with HLA-C indicating NK cell-mediated immune pressure involving KIR2DL^+^ NK cells (36, 37). It is therefore conceivable that binding affinities predetermined by *KIR2DL/HLA-C* genotypes may have a similar impact.

In this context, expansion of KIR^+^ NK cell subsets can be an indicator for the involvement of specific KIR^+^ NK cell subsets in the antiviral immune response against HIV-1 (58–60). HIV-1 infection has been associated with significant changes in the NK cell receptor repertoire (61–63). In our phenotypical characterization we investigated the expression of NK cell receptors interacting with various HLA class I molecules in a cohort of untreated, viremic HIV-1^+^ individuals. Noteworthy, decreased numbers of CD8^+^ NK cells in HIV-1-infected individuals have been observed in a previous study, where it correlated positively with HIV loads and inversely with CD4^+^ T cell counts (64). We also observed an overall contraction of the NKG2A^+^ NK cell subset which was closely tied to an expansion of NKG2C^+^ and CD57^+^ NK cells in the same donors. The inhibitory NKG2A receptor interacts with the non-classical HLA class I molecule HLA-E on target cells, preventing healthy cells from NK cell-mediated lysis. Additionally, HLA-E is recognized by NKG2C, which delivers an activating signal to the NK cell (65). NKG2A^+^ NK cells have been implicated to possess superior anti-HIV activity compared to other subsets, whereas KIR2DL^+^ NK cells had a diminished ability to degranulate in response to HIV-1-infected CD4^+^ T cells even in the absence of NKG2A (66). In line with our results, Mavilio et al., showed a decreased expression of NKG2A in HIV-1-infected viremic individuals, which was associated with reduced inhibitory function in a redirected killing assay (61). Loss of NKG2A^+^ NK cells corresponded with a high expansion of NK cells expressing NKG2C (67). In contrast, higher NKG2A expression levels in cytotoxic NK cell subsets have been reported in individuals with late stage HIV-1 infection (68). Expansion of NKG2C^+^ and CD57^+^ NK cells is not unique to HIV-1 infection (69). Similar changes have been observed for CMV and Hantavirus infection (70–72). It is still under investigation if these CD57^+^NKG2C^+^ NK cells have memory-like features specific for viral infections (73). Data on CMV seropositivity was not available for this cohort and therefore we cannot rule out a CMV-associated expansion in the HIV-1^+^ cohort. However, an earlier study of our group indicated a CMV-independent, HIV-1-driven expansion of KIR2DL^+^ NK cells (32).

Analysis of KIR^+^ NK cells recognizing HLA-C initially showed a decrease of cells expressing the activating receptor KIR2DS4, while percentage of most inhibitory receptors remained unchanged. KIR2DS4 binds a subset of HLA-C1 and -C2 allotypes and HLA-A*11 (18) however its role in disease progression and NK cells regulation is not fully understood. KIR2DS4 has been associated with high viral loads and promotion of HIV-1 pathogenesis in chronic HIV-1 infection, probably through excessive NK cell activation (74, 75). HIV-1^+^ individuals showed a higher frequency of the inhibitory KIR3DL2 receptor, which recognizes only HLA-A3 and -A11 in a peptide specific manner (11) and HLA-B27 (76). So far, not much is known about the role of KIR3DL2 in HIV-1 infection but other studies showed also an increased expression of KIR3DL2 in chronic HCV patients (77) and on activated NK cells from patients with spondylarthritis (78). Further stratification of our data based on *HLA-C* and *KIR2DL* genotypes indicated that the HIV-1-associated changes in the proportion of KIR2DL^+^ NK cells within the NK cell pool were predetermined by host genetics. The frequency of KIR2DL1^+^ NK cells was linked to the number of the cognate *HLA-C2* alleles, which we showed previously for individuals with primary HIV-1 infection (32). The increased frequency is potentially related to an increased surface expression of the respective HLA-C2 ligand. In addition, differentiation of *KIR2DL1* alleles displayed a similar “gene-dose” effect for the analyzed *KIR2DL1* alleles with the exception of KIR2DL1*001/002. Further analysis showed a positive correlation between KIR2DL/HLA-C binding affinities and the frequency of the respective KIR2DL^+^ NK cell subset, thus further corroborating our hypothesis. However, we cannot rule out that the differences in the relative frequency of NK cells expressing various *KIR2DL* allotypes is due to changes in non-coding regions that may impact expression on an individual cell and within an individual NK cell pool (79–81).

Lastly, we investigated the impact of KIR/HLA-C genotypes on Vpu sequence polymorphisms. Molecular characterization of Vpu sequences of primary viruses identified five amino acid positions (3, 5, 15, 16 and 24) in the transmembrane and extracellular domain of Vpu that are associated with HLA-C downregulation (30). Grouping the amino acid position and residues based on their *HLA-C* genotype showed no differences in the frequency of amino acid positions/residues that downregulate HLA-C. We further tested whether differential *HLA-C* allele expression or HLA-C/KIR2DL binding were associated with AA residues that are involved in HLA-C downmodulation (28, 30). We observed an increased frequency of glycine or threonine at AA position 16 in individuals with stronger HLA-C/KIR2DL binding combinations compared to other amino acid residues at position 16, hinting that the binding affinities between KIR2DL and HLA-C molecules might be an additional factor impacting Vpu sequence polymorphisms. It should be noted that the observed signals contain a certain level of uncertainty that are attributed to the numerous allotype combinations in the cohort and the ambiguity of *KIR* allele typing. In general, the enormous diversity in HLA-C/KIR2DL binding combinations and different affinities, influencing the activation of NK cells and immune evasion mechanisms of HIV-1, make it challenging to develop a prediction model for specific Vpu sequence polymorphisms and consequences for NK cell activation based on *HLA-C/KIR2DL* genotypes.

Altogether, this study demonstrates the significant effects of HIV-1 infection on the NK cell pool in viremic, untreated HIV-1^+^ individuals and provides evidence that the specific changes in the KIR2DL repertoire are predetermined by the underlying *KIR2DL/HLA-C* genotypes. The results also make a case that high resolution *KIR* typing and the generation of KIR/HLA binding models may be warranted to improve disease models and to potentially predict NK cell-associated immune responses and disease progression in various pathological settings and subsequently NK cell-based immune therapies.

## Data Availability Statement

Sequence data presented in the study are deposited in the European Nucleotide Archive (ENA), accession number #PRJEB53333. Storage of other raw data is performed by the Leibniz Institute of Virology on an internal server. Raw data will be made available upon request and can be shared after confirming that data will be used within the scope of the originally provided informed consent.

## Ethics Statement

For this study, residual amounts of anonymized peripheral blood samples were used which were routinely taken from healthy blood donors at the the Institute for Transfusion Medicine, University Medical Center Hamburg-Eppendorf, Hamburg, Germany, and would have been discarded otherwise. All blood donors gave their general written consent to usage of their blood samples for scientific studies in an anonymized form. The anonymized use of human material complies with a vote by the ethics committee of the German Medical Association. Healthy blood donors recruited at the University Medical Center Hamburg-Eppendorf, Hamburg, Germany, provided written informed consent and studies were approved by the ethics committee of the Ärztekammer Hamburg (PV4780). Donors recruited through the Translational Platform HIV (TP-HIV) Cohort by the German Center for Infection Research (DZIF) provided written informed consent and the study was approved by the ethics committee of the Ärztekammer Hamburg (MC-316/14).

## Author Contributions

Conceptualization: CK; Funding acquisition: CK, MA, and AH; Methodology: CK, SVo, AN, and AH. Validation: SVo and AL; Formal Analysis: CK, SVo, SVi, and LR; Investigation: SVo, AL, TT, SB, DI, and JN; Resources: SVo, AN, PF, MA, GS, SP, GMNB, CL, AM, RP, NP, JR, SS, CS, CDS, EW, CW, PJN, JS, and AHS; Writing – original draft: CK and SVo; Writing - review and editing: all authors; Visualization: CK and SVo; Supervision: CK. All authors contributed to the article and approved the submitted version.

## Funding

The study was supported by the German Research foundation (DFG) (KO5139/3-1) and the German Center for Infection Research (DZIF) (TTU 04.820_01). TT and SB received funding by the state of Hamburg, Germany (LFF-FV74 and LFF-FV78). JR received funding by the German Center for Infection Research (DZIF) (TTU HIV 04.817, 04.819, 04.919) and the Else-Kröner-Fresenius Stiftung (EKFS) (2021_EKEA.25). AH was supported through the Clinician Scientist Program of the Faculty of Medicine, University Medical Centre Hamburg-Eppendorf.

## Conflict of Interest

CDS reports grants and personal fees from AbbVie, grants, fees and non-financial support from Gilead Sciences, grants and personal fees from Janssen-Cilag, grants and personal fees from MSD, grants from Cepheid, personal fees from GSK, grants and personal fees from ViiV Healthcare, during the conduct of the study; fees from AstraZeneca, other from Apeiron, grants, personal fees and non-financial support from BBraun Melsungen, grants, personal fees from BioNtech, personal fees from Eli Lilly, personal fees from Formycon, personal fees from Molecular partners, grants and personal fees from Eli Lilly, personal fees from Roche, personal fees from SOBI.

The remaining authors declare that the research was conducted in the absence of any commercial or financial relationships that could be construed as a potential conflict of interest.

## Publisher’s Note

All claims expressed in this article are solely those of the authors and do not necessarily represent those of their affiliated organizations, or those of the publisher, the editors and the reviewers. Any product that may be evaluated in this article, or claim that may be made by its manufacturer, is not guaranteed or endorsed by the publisher.
