# Supplementary material for: Host KIR/HLA-C Genotypes Determine HIV-Mediated Changes of the NK Cell Repertoire and Are Associated With Vpu Sequence Variations Impacting Downmodulation of HLA-C
Source: Front Immunol. 2022 Jul 15;13:922252. doi: 10.3389/fimmu.2022.922252 (PMC9334850; doi:10.3389/fimmu.2022.922252)
Supplement: Supplementary file 1 [file DataSheet_1.docx]

**Supplementary Material**


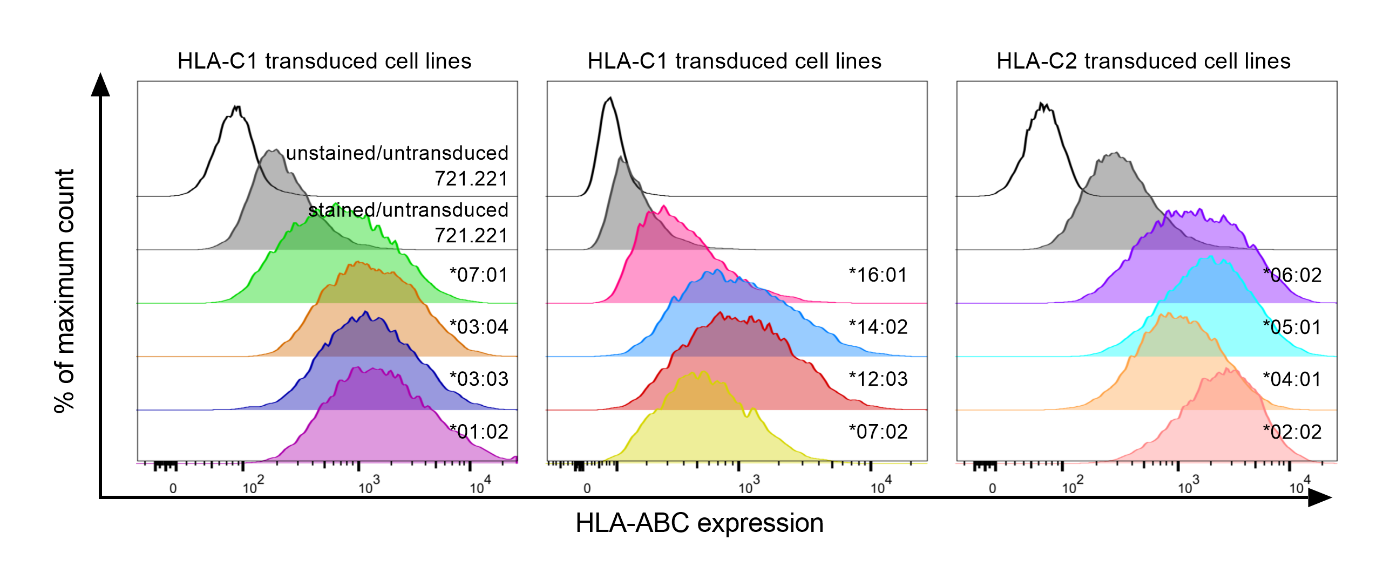


**Supplementary Figure 1 |** **HLA-ABC expression of untransduced and HLA-C transduced 721.221 cell lines**. All three histograms compare the HLA-ABC expression measured by flow cytometry (clone W6/32) of HLA-C transduced 721.221 cell lines to unstained (black, not filled) and stained (grey, filled) untransduced 721.221 cells. The left and middle histograms show the expression of the eight HLA-C1 transduced cell lines (HLA-C*01:02, *03:03, *03:04, *07:01, *07:02, *12:03, *14:02 and *16:01). The right histogram shows the HLA-C expression of the generated HLA-C2 cell lines (HLA-C*02:02; *04:01, *05:01 and *0602).


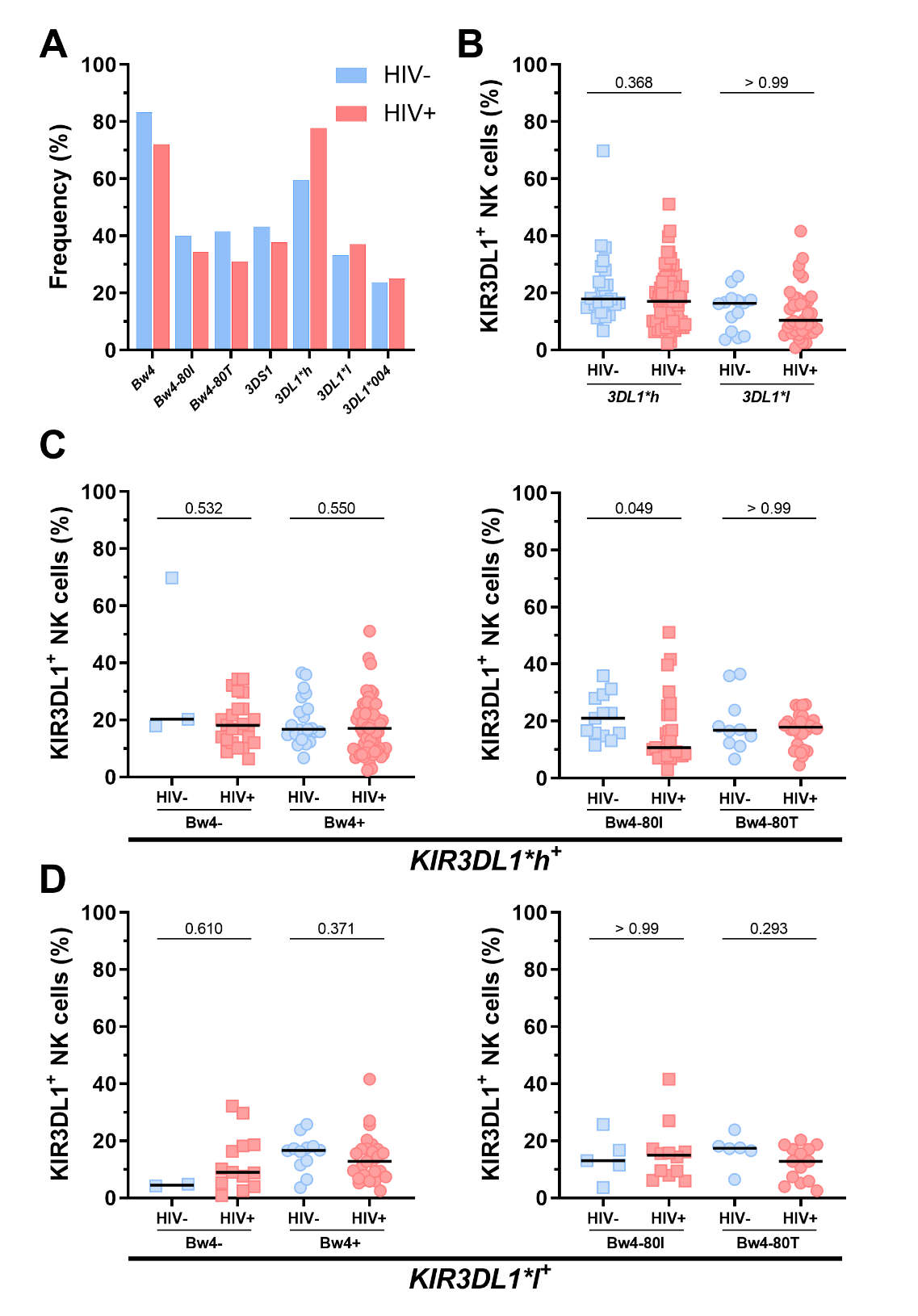


**Supplementary Figure 2 | Stratification of KIR3DL1 allotype groups. (A)** Frequency of *HLA-B Bw4* subtypes, *KIR3DS1*, *KIR3DL1*h* (high expressed alleles: *001, *002, *008, *009 and *015), *KIR3DL1*l* (low expressed alleles: *005, *007) and *KIR3DL1*004* alleles in HIV-1^-^ (blue) and HIV-1^+^ individuals (red). **(B)** Scatter plots display the percentage of KIR3DL1^+^ bulk NK cells stratified by *KIR3DL1*h* and *KIR3DL1*l* alleles. **(C)** Scatter plots display the percentage of KIR3DL1^+^ bulk NK cells of donors with *KIR3DL1*h* alleles stratified by Bw4 subtypes. **(D)** Scatter plots display the percentage of KIR3DL1^+^ bulk NK cells of donors with *KIR3DL1*l* alleles stratified by Bw4 subtypes. (B-D) Mann-Whitney test. p values were adjusted for multiple comparisons (Bonferroni).


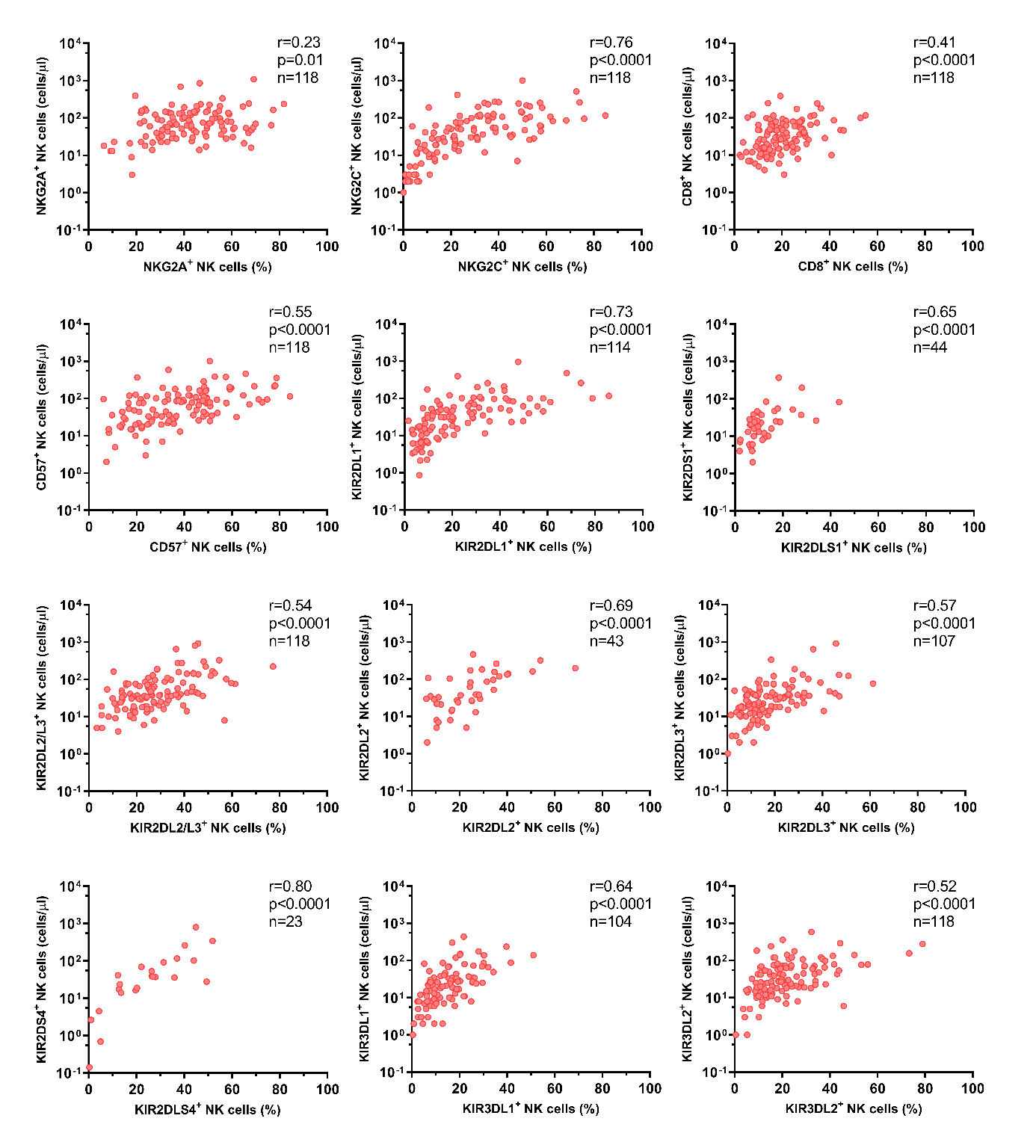


**Supplementary Figure 3 | Cell numbers of NK cell subpopulations correlate positively with relative frequency of the respective receptor^+^ NK cells.** Correlation between percentage of receptor^+^ NK cells and absolute cell numbers of NK cells expressing the receptors NKG2A, NKG2C, CD57, CD8, KIR2DS1, KIR2DL1, KIR2DL2/L3, KIR2DL2, KIR2DL3, KIR2DS4, KIR3DL1 and KIR3DL2 in HIV-1^+^ individuals. Spearman rank correlation analysis. p values were adjusted for multiple comparisons (Benjamini/Krieger/Yekutieli).


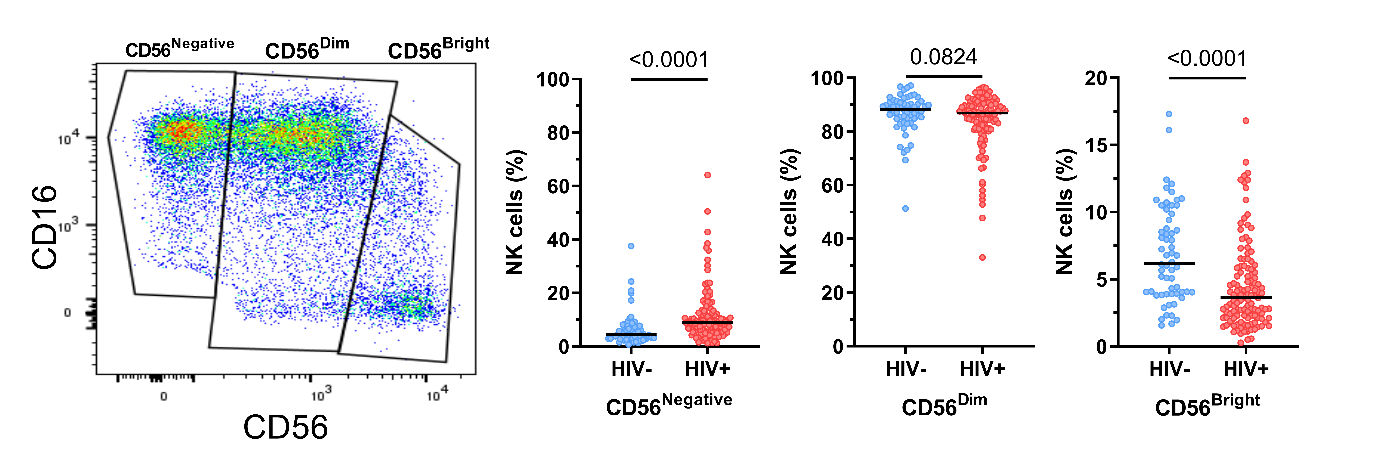


**Supplementary Figure 4** **| HIV-1 infection is associated with changes of the CD56 NK cell subset distribution.** Exemplary flow cytometry plot showing gating of CD56^Negative^, CD56^Dim^, and CD56^Bright^ NK cells. Dot plots displaying the relative frequency of CD56^Negative^, CD56^Dim^, and CD56^Bright^ cells (left to right) within bulk NK cells from HIV-1^-^ (n = 60) and untreated HIV -1^+^ (n = 122) individuals. Bars indicate the median for each group. Mann-Whitney test was used to determine differences between HIV-1^-^ and HIV-1^+^ individuals.


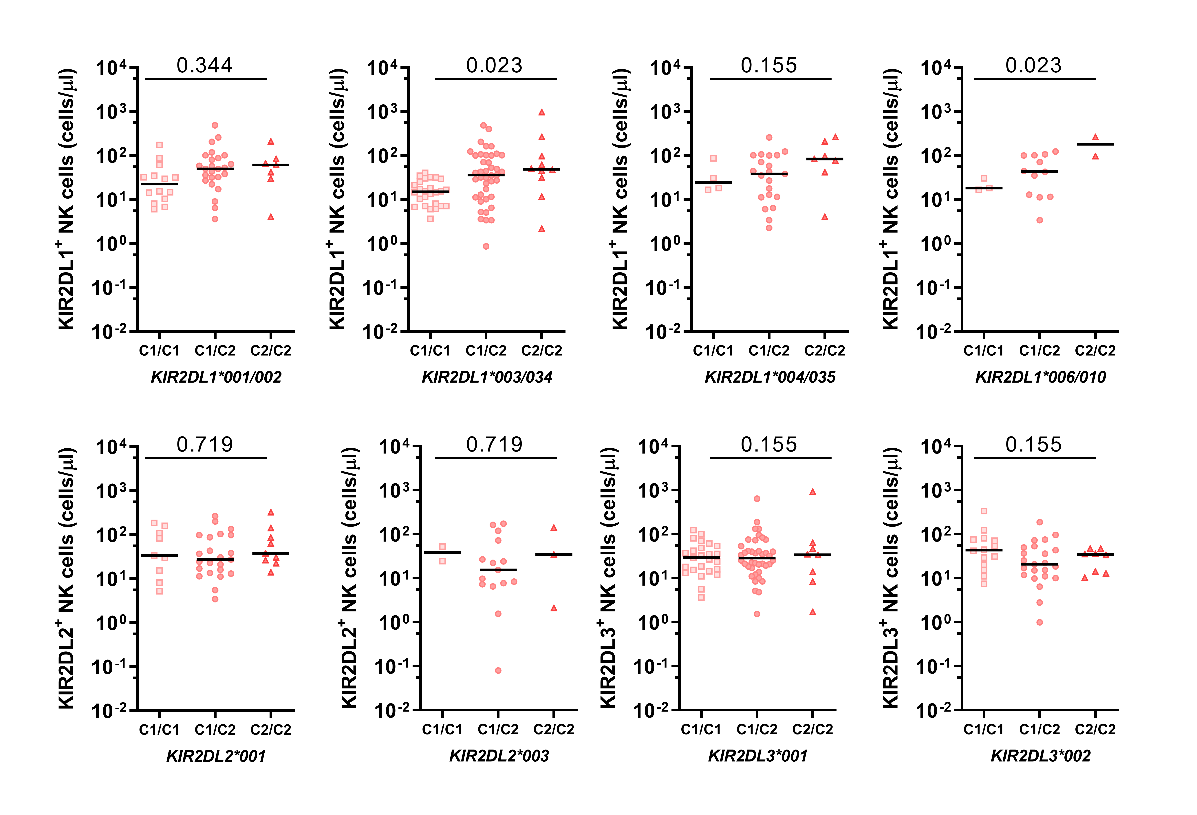


**Supplementary Figure 5** **| Cell number of KIR2DL1^+^, KIR2DL2^+^ and KIR2DL3^+^ NK cells in HIV-1^+^ individuals.** KIR2DL1^+^, KIR2DL2^+^ and KIR2DL3^+^ NK cells of HIV-1^+^ individuals were stratified by HLA-C group genotype and KIR2DL alleles. One-way ANOVA, test for linear trend was used to determine differences between HLA-C group genotypes. P values were adjusted for multiple comparisons (Benjamini/Krieger/Yekutieli).

**Supplementary Table 1: Descriptive statistics for all eleven analyzed NK cell receptors.**

|  | HIV- | HIV+ | HIV- | HIV+ | HIV- | HIV+ |  |  |
| --- | --- | --- | --- | --- | --- | --- | --- | --- |
| **receptor** | **n** | **n** | **median (25%/75% percentile)** | **median (25%/75% percentile)** | **Min/Max** | **Min/Max** | **p value*** | **adjusted p value*** |
| NKG2A | 60 | 122 | 54.4  (45.28/64.25) | 42.50  (31.65/53.48) | 17.2/81.8 | 6.44/81.9 | <0.0001 | <0.0001 |
| NKG2C | 60 | 122 | 3.47 (1.63/5.54) | 23.45 (11.03/84.8) | 0.1/56.3 | 0.1/84.8 | <0.0001 | <0.0001 |
| CD8 | 60 | 122 | 32.3 (24.58/42.2) | 18.9 (13.58/26.13) | 5.74/57.9 | 2.34/55.1 | <0.0001 | <0.0001 |
| CD57 | 60 | 122 | 31.5 (20.2/42.33) | 36.7 (23.55/49.25) | 8.85/63.6 | 6.14/84.4 | 0.17 | 0.20 |
| KIR2DL1 | 59 | 118 | 15.0 (12.4/19.4) | 17.0 (9.22/31.75) | 3.55/56.7 | 1.6/85.8 | 0.07 | 0.12 |
| KIR2DS1 | 26 | 47 | 14.75 (10.6/19.43) | 9.86 (6.98/17.1) | 6.18/34.6 | 0.04/43.6 | 0.23 | 0.20 |
| KIR2DL2/L3 | 60 | 122 | 27.0 (19.35/32.23) | 26.3 (18.7/37.35) | 12.9/67.6 | 3.3/77.3 | 0.52 | 0.41 |
| KIR2DL2 | 28 | 64 | 12.1 (7.49/18.73) | 17.5 (10.63/28.5) | 0.1/32.9 | 0.1/68.7 | 0.24 | 0.20 |
| KIR2DL3 | 55 | 111 | 20.7 (13.7/23.9) | 15.3  (9.6/24.4) | 8.2/41.3 | 0.3/61.3 | 0.09 | 0.12 |
| KIR2DS4 | 19 | 25 | 41.6 (29.30/45.30) | 26.5 (12.85/38.6) | 0.06/63.8 | 0.3/60.7 | 0.021 | 0.049 |
| KIR3DL1 | 55 | 108 | 16.2 (11.1/23.9) | 13.25 (7.8/19.68) | 0.2/69.8 | 0.1/51.0 | 0.24 | 0.20 |
| KIR3DL2 | 60 | 122 | 13.4  (7.83/19.2) | 19.65  (12.48/28.75) | 2.96/34.7 | 0.48/78.9 | 0.037 | 0.070 |
| * Multiple linear regression analysis (HIV, sex, age), adjustment for multiplicity (Benjamini, Krieger and Yekutieli). | | | | | | | | |
